# Simultaneous isolation of hormone receptor–positive breast cancer organoids and fibroblasts reveals stroma-mediated resistance mechanisms

**DOI:** 10.1016/j.jbc.2023.105021

**Published:** 2023-07-07

**Authors:** Jenny M. Hogstrom, Kayla A. Cruz, Laura M. Selfors, Madelyn N. Ward, Tejas S. Mehta, Naama Kanarek, Jordana Philips, Vandana Dialani, Gerburg Wulf, Laura C. Collins, Jaymin M. Patel, Taru Muranen

**Affiliations:** 1 Department of Medicine, Beth Israel Deaconess Medical Center, Harvard Medical School, Boston, Massachusetts, USA; 2 Department of Cell Biology, Harvard Medical School, Boston, Massachusetts, USA; 3 Department of Radiology, Beth Israel Deaconess Medical Center, Harvard Medical School, Boston, Massachusetts, USA; 4 Department of Pathology, Boston Children’s Hospital, Harvard Medical School, Boston, Massachusetts, USA; 5 Department of Pathology, Beth Israel Deaconess Medical Center, Harvard Medical School, Boston, Massachusetts, USA

**Keywords:** patient-derived organoids, PDO, cancer-associated fibroblast, CAF, drug resistance, hormone receptor–positive breast cancer, luminal breast cancer, estrogen receptor, tumor-stroma cross talk, cytokine, chemokine, GROα, CCL19, fulvestrant, co-culture model

## Abstract

Recurrent hormone receptor–positive (HR+) breast cancer kills more than 600,000 women annually. Although HR+ breast cancers typically respond well to therapies, approximately 30% of patients relapse. At this stage, the tumors are usually metastatic and incurable. Resistance to therapy, particularly endocrine therapy is typically thought to be tumor intrinsic (*e.g.*, estrogen receptor mutations). However, tumor-extrinsic factors also contribute to resistance. For example, stromal cells, such as cancer-associated fibroblasts (CAFs), residing in the tumor microenvironment, are known to stimulate resistance and disease recurrence. Recurrence in HR+ disease has been difficult to study due to the prolonged clinical course, complex nature of resistance, and lack of appropriate model systems. Existing HR+ models are limited to HR+ cell lines, a few HR+ organoid models, and xenograft models that all lack components of the human stroma. Therefore, there is an urgent need for more clinically relevant models to study the complex nature of recurrent HR+ breast cancer, and the factors contributing to treatment relapse. Here, we present an optimized protocol that allows a high take-rate, and simultaneous propagation of patient-derived organoids (PDOs) and matching CAFs, from primary and metastatic HR+ breast cancers. Our protocol allows for long-term culturing of HR+ PDOs that retain estrogen receptor expression and show responsiveness to hormone therapy. We further show the functional utility of this system by identifying CAF-secreted cytokines, such as growth-regulated oncogene α , as stroma-derived resistance drivers to endocrine therapy in HR+ PDOs.

Globally ∼2.1 million women are diagnosed with breast cancer yearly. The majority (∼70%) of newly diagnosed breast cancers are hormone receptor–positive (HR+), expressing estrogen receptor (ER) with or without progesterone receptor (1). Despite available treatment options more than 30% of HR+ breast cancer patients are expected to recur. At recurrence the tumors are usually metastatic and resistant to standard-of-care therapies (2, 3). The standard-of-care in HR+ breast cancer are targeted therapies, such as ER-targeting (*e.g.* tamoxifen and fulvestrant), aromatase inhibition, or inhibitors of cyclin-dependent kinases 4/6 (CDK4/6), or a combination of these treatments (4, 5, 6, 7, 8). Additional targeted therapies exist that could be used (9, 10, 11, 12), but clinical trial testing is limited by a lack of predictive biomarkers and clinically relevant model systems. This lack of relevant model systems has made HR+ breast cancer difficult to study. Existing models for HR+ disease include the established HR+ breast cancer cell lines, such as MCF7 and T47D, which although a valuable tool, do not faithfully replicate many of the patients’ features such as drug responses (13).

Patient-derived organoids (PDOs) are 3-dimensional structures with organ-like features grown in matrix-like media from a single patient’s tumor specimen (14), which also more closely resemble the original tumor’s features. Historically, growing HR+ PDOs has been a challenge. A method for deriving breast tumor organoids, including HR+ organoids, was published in 2018 (15). Unfortunately, replicating the success rate of that study and growing HR+ PDOs has been challenging to the field, with a low take-rate (10%), the PDOs losing their HR expression and ceasing to grow after only a few passages (16, 17). A recent study described a method for growing breast tumor organoids propagated from patient-derived xenografts (PDX) (18). The study established a large collection of primary and metastatic patient-derived xenograft organoids (PDxO), including HR+ PDxOs, with a take-rate of 9% for primary tumors and 16% for metastatic tumors. Currently, there are only a few models of metastatic HR+ breast cancer available (18, 19). Establishing novel models of HR+ primary and metastatic breast cancer would greatly advance research on disease relapse and drug resistance.

Another aspect of the tumor biology, the tumor microenvironment (TME), such as cancer-associated fibroblasts (CAFs), that significantly impact tumor biology and resistance to therapies, are rarely incorporated into studies of breast cancer recurrence in patient-derived models. CAFs are known to induce resistance to targeted therapies and modify the TME in breast cancer (20, 21, 22, 23, 24, 25, 26). For example, CAFs drive resistance to HER2-targeted therapies (24, 26, 27, 28), suppress apoptotic programs (24), promote chemoresistance (29), support tumor stem cells (30), and reduce effectiveness of ER-targeting therapies (24, 25, 31). CAFs also secrete proteins that alter the TME, making it physically stiffer, which in turn stimulates more aggressive tumor cell behavior (22). Furthermore, CAFs secrete immunomodulatory cytokines that can impact immune cell function. Both, matrix proteins and cytokines can induce resistance to breast cancer therapies (32, 33, 34, 35). Despite the CAFs contribution to all these aspects of tumor biology, they are rarely incorporated into studies on drivers of treatment resistance, and very few protocols include them in PDO studies.

In this study, we set out to develop patient-derived model systems for HR+ breast cancer that would also incorporate elements of the TME. We also built an improved method for deriving HR+ PDOs, both from primary site, as well as metastatic loci with ∼50% take-rate. Importantly, our method allows isolation and passaging of HR+ bone metastasis, which historically have been difficult to grow, with only ∼2 to 3 established PDX or PDxO lines available. As a proof of principle, we have used these models, and these data have revealed cross talk between PDOs and CAFs that stimulate treatment resistance and identified growth-regulated oncogene α (GROα) and chemokine (C-C motif) ligand 19 (CCL19) as drivers of endocrine therapy resistance.

## Results

### PDO isolation from tissue samples

**Figure 1:**
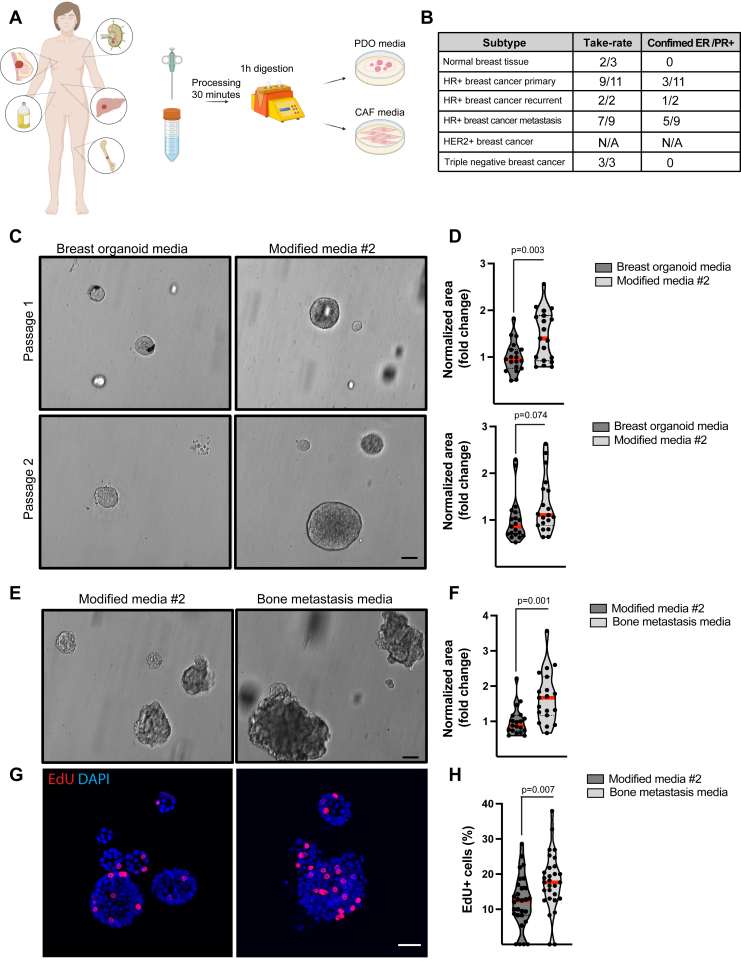
**Optimized media supports growth of hormone receptor–positive patient-derived organoid cultures.***A*, schematic illustration of processing of PDOs and CAFs from tumor. *B*, take-rate and confirmed ER/PR positivity of established PDO lines. *C* and *D*, representative brightfield images (*C*) and quantification of diameter (*D*) in patient #27 PDOs grown in breast organoid media and modified media #2 at passage 2. *E* and *F*, representative brightfield images (*E*) and quantification of diameter (*F*) in patient #26 PDOs grown in modified media #2 and bone metastasis media at passage 2. *G* and *H*, representative confocal images (*G*) and quantifications (*H*) of 5-ethynyl-2′-deoxyuridine (EdU) positive cells (*red*) in PDOs grown in modified media #2 and bone metastasis media at passage 2. The scale bars represent 200 μm (*C* and *E*), 40 μm (*G*). Proliferation was assessed by pulsing PDOs with EdU for 4 h and quantifying the ratio of EdU+ cells per total (DAPI+) number of cells in 20 to 30 PDOs from one experiment. Each data point represents the percentage of EdU+ cells in one PDO, *red line* indicates the mean ratio of EdU+ cells per treatment group. The diameter of PDOs was measured using CellProfiler software and normalized to breast organoid media (*D*) or modified media #2 (*F*). Student’s *t* test was used to assess significance. CAFs, cancer-associated fibroblasts; DAPI, 4′,6-diamidino-2-phenylindole; ER, estrogen receptor; PDO, patient-derived organoids; PR, progesterone receptor.

In accordance with principles of Good Clinical Practice and Declaration of Helsinki a prospective tissue protocol was established to collect additional breast biopsies during routine scheduled procedures for patients with known history or clinical concern for breast cancer. The protocol included 28 patients, and patient samples reported were collected between January 2020 and November 2021. Screening for potential patients was performed by subinvestigators specializing in breast radiology, surgery, or medical oncology. Standard imaging modality used to identify target breast lesions included mammography, ultrasound, and/or MRI while imaging for metastatic lesions included computed tomography (CT) imaging with contrast, [^18^F] Fludeoxyglucose PET/CT or MRI. Pertinent clinical information such as patient demographics, pathology results from diagnostic biopsy and/or surgical resection, molecular and genomic findings, clinical stages, treatment plans, and response/recurrence were collected in stored in REDCap database (36, 37) (Table S1, Sheets 1–2). Tissue was collected by core-needle biopsy (CNB) or fine-needle aspiration (FNA) biopsy with at least 2 to 4 specimens per tumor. Tissue specimens were placed in deidentified vial with alpha-numeric ID and transferred in cold PBS within 30 to 60 min for further processing in protocol designated research laboratory. Specimens were immediately processed by enzymatic digestion to establish PDO cultures (Fig. 1*A*). Any pleural fluid samples were embedded in basement membrane extract (BME). The take-rate was measured as three viable passages over 4 to 6 weeks. The overall initial take-rate for all the PDO cultures was 82% (Fig. 1*B*).

**Figure 2:**
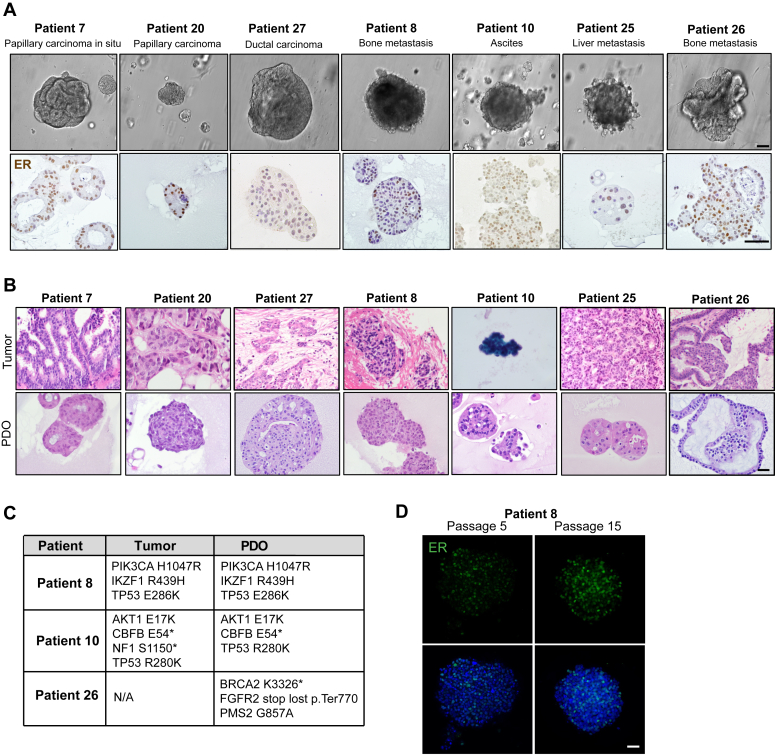
**Patient-derived organoid cultures retain estrogen receptor (ER) expression during long-term culture and resemble original patient tumors histology.***A*, representative brightfield images and ER immunohistochemistry of established HR+ PDO lines. *B*, brightfield images of H&E stained matching patient tumor and PDOs. *C*, table showing mutations found in the original patient tumors and at matching PDOs isolated from these tumors. Patient #8 and #10 had prior mutational analysis performed in their tumors. PDOs were exome sequenced to identify matching mutations. *D*, representative confocal images of patient #8 PDOs stained for ER (*green*) and nuclei (DAPI: *blue*) at passage 5 and passage 15. The scale bars represent 200 μm (*A*), 100 μm (*B*), and 40 μm (*D*). DAPI, 4′,6-diamidino-2-phenylindole; ER, estrogen receptor; HR+, hormone receptor–positive; PDOs, patient-derived organoids.

To optimize the culturing conditions for HR+ PDOs, we assessed the growth of primary HR+ PDOs in two modified versions of breast organoid media (15). We first removed R-spondin 3, noggin, neuregulin and B27 supplement and added 17-β-estradiol. However, this media resulted impaired growth and 2D adhesion of the PDOs (Fig. 1, *A* and *C*: modified media #1). We next added hydrocortisone and 17-β-estradiol to the published media (15), and this resulted in larger PDOs and increased proliferation (Figs. 1, *C* and *D* and S1, *A*–*D*: modified media #2). We further optimized the media to support growth of bone metastasis samples by adding growth factors that are present in the bone microenvironment (C-X-C motif chemokine ligand 12 (CXCL12), osteopontin, and insulin-like growth factor 1) (38), which significantly increased the growth of the bone metastasis PDOs (Figs. 1, *E*–*H* and S1*E*). All HR+ PDOs were inspected for ER expression after three passages since this is often rapidly lost in cultured PDOs. Our collection protocol was able to retain ER expression in approximately half of the initially established HR+ PDOs bringing the overall take-rate for the HR+ PDOs to ∼40% (Figs. 1*B* and 2*A*). We further confirmed this ER expression over time (Figs. 2*D* and S2, *B* and *C*) and noted that the metastatic PDOs retained their ER expression over 20 passages and over 1 year. We also compared the PDO histology and ER expression to that of the original tumor and noted that the PDOs had similar architectural and cytologic features, such as cribriforming or gland formation and nuclear membrane irregularities, and ER expression levels resembling those of the original patient tumors (Figs. 2*B* and S2*A*). Finally, to further confirm that the PDOs were of breast tumor origin, and harbored breast cancer-associated mutations, we performed exome sequencing in three of our PDO lines (patients #8, #10, and #26) at passage 25, 30, and 10, respectively, and validated that PDOs #8 and #10, which had sequencing data available for the primary tumor samples, harbored the same mutations as the primary patient tumor. Although we did not have sequencing data available from the original tumor of patient #26, the #26 PDO had mutations typically present in breast cancer, such as mutations in *BRCA2*, *FGFR2*, and *PMS2* (Fig. 2*C*).

### CAF isolation and characterization

**Figure 3:**
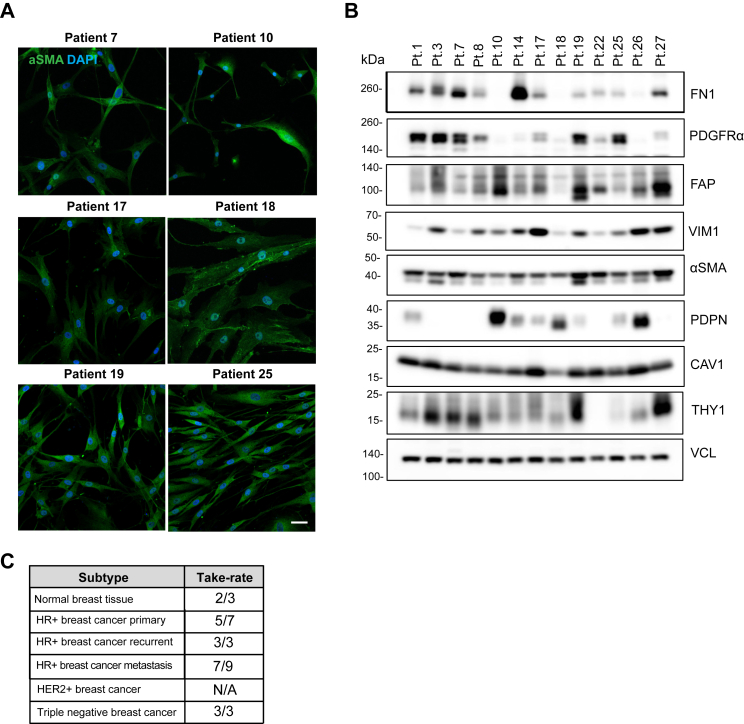
**Propagation of cancer-associated fibroblasts from primary and metastatic ER+ breast cancer.***A*, confocal images of the CAF lines, stained for the CAF marker alpha-smooth muscle actin (αSMA: *green*) and nuclei (DAPI: *blue*). *B*, Western blot analysis of CAF markers: fibronectin (FN1), platelet-derived growth factor alpha (PDGFRα), fibroblast activating protein (FAP), vimentin (VIM1), αSMA, podoplanin (PDPN), caveolin-1 (CAV1) and Thy-1 cell surface antigen (THY1). Vinculin (VCL) was used as loading control. *C*, total numbers of CAF lines and the successful take-rate from biopsies. The scale bar represents 40 μm. CAF, cancer-associated fibroblasts; DAPI, 4′,6-diamidino-2-phenylindole; ER, estrogen receptor.

Our tissue collection protocol included simultaneous isolation of stromal cells/CAFs, from the same CNBs, allowing us to generate matching PDO–CAF pairs. We were successful at isolating the CAFs from multiple locations, including the primary site and metastatic loci (the lymph node, ascites, the bone, and the liver) (Fig. S3, Table S1). The overall take-rate for the CAFs was 64% (Fig. 3*C*). Since the CAF morphology varied depending on the CAF line and isolation site, we further characterized the CAFs by assessing the expression of multiple CAF markers (fibronectin (FN1), platelet-derived growth factor alpha (PDGFRα), podoplanin (PDPN), fibroblast activating protein (FAP), vimentin (VIM), alpha smooth muscle actin (αSMA), caveolin-1 (CAV1), and thy-1 cell surface antigen (THY1)) (Fig. 3, *A* and *B*). We observed CAF-to-CAF variability in the marker expression, as is expected due the CAF heterogeneity (39), and isolation from different loci. Since serial passaging of nontransformed fibroblasts induce senescence (39), all the experiments described in this manuscript were performed using CAFs of less than ten passages.

### Analysis of PDO drug responses

**Figure 4:**
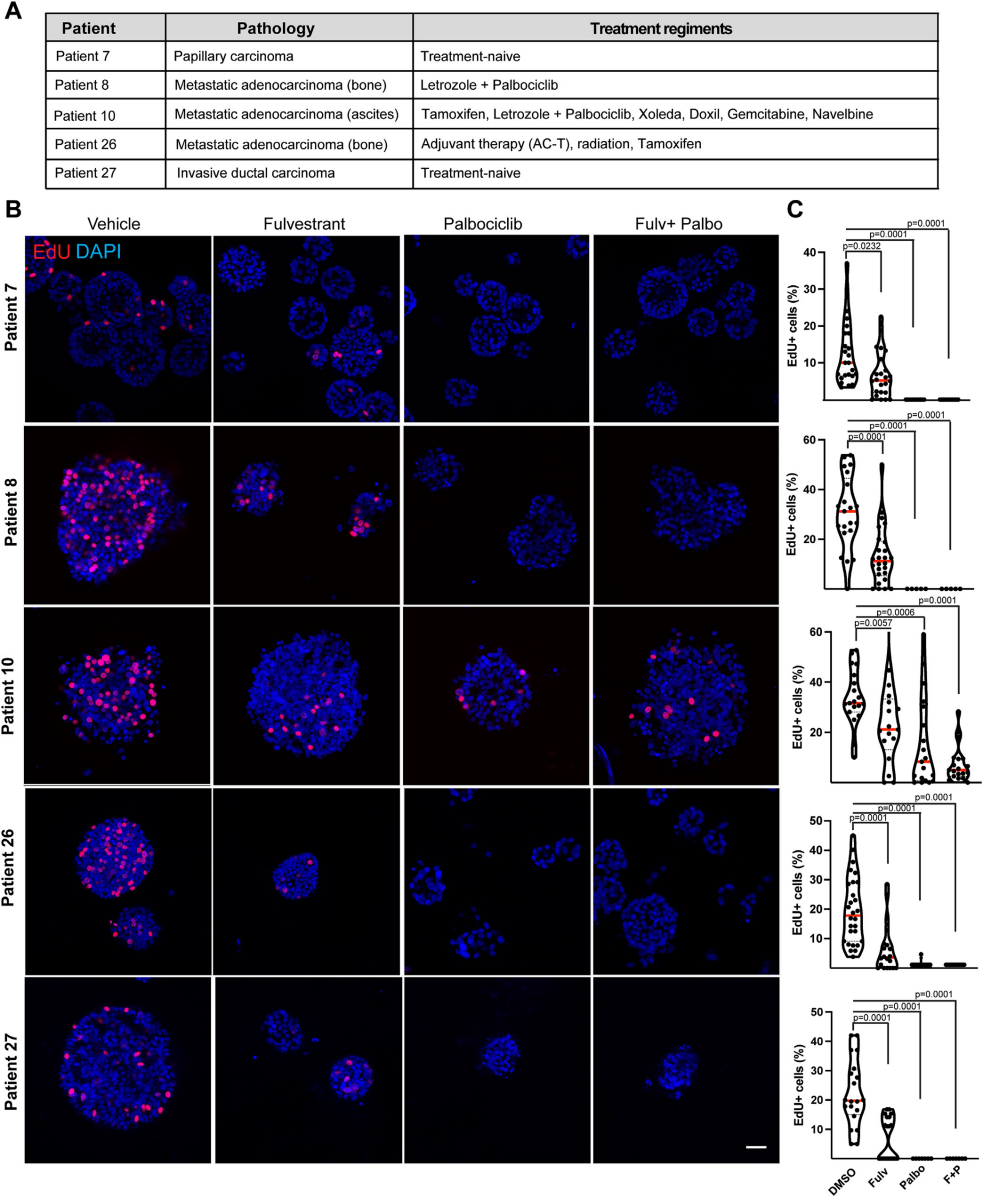
**Patient-derived organoid cultures regain partial sensitivity to targeted therapies in culture.***A*, pathology and treatment regiments of indicated patients. *B* and *C*, representative confocal images (*B*) and quantifications (*C*) of 5-ethynyl-2′-deoxyuridine (EdU) positive cells (*red*) in PDOs treated with vehicle, 500 nM fulvestrant, 1 μM palbociclib, or combination of fulvestrant and palbociclib for 96 h. The scale bar represents 40 μm. Proliferation was assessed by pulsing PDOs with EdU for 4 h and quantifying the ratio of EdU+ cells per total (DAPI+) number of cells in 20 to 30 PDOs from one experiment (#7 and #27) or three independent experiments (#8, #10, and #26). Each data point represents the percentage of EdU+ cells in one PDO, *red line* indicates the mean ratio of EdU+ cells per treatment group. Student’s *t* test was used to assess significance comparing each treatment group to vehicle sample. DAPI, 4′,6-diamidino-2-phenylindole; EdU, 5-ethynyl-2′-deoxyuridine; PDOs, patient-derived organoids.

Among the collected patient characteristics, treatment history and responses in the relapsed cases were included (Fig. 4*A*, Table S1). All HR+ recurrent cases were treated with ER-targeting agents (fulvestrant, tamoxifen, and letrozole), or CDK4/6 targeting therapies (abemaciclib, palbociclib, and ribociclib). To investigate how the HR+ PDOs respond to treatment regimens used in the patients and if they respond similarly to the original tumor, we treated five HR+ PDO lines (#7, #8, #10, #26, and #27) with an ER-targeting agent (fulvestrant), or a CDK4/6 targeting agent (palbociclib), or with the combination (Fig. 4, *B* and *C*). PDOs were plated in Cultrex and after 2 days drugs were added for 4 days (fulvestrant 500 nM and/or palbociclib 1 μM). On the day of fixation, 5-ethynyl-2′-deoxyuridine (EdU) was added to the cultures for 4 h to label proliferating cells, the PDOs were fixed and stained for EdU, or Ki67 (cell proliferation marker) and p21 (cell cycle inhibition marker) and counterstained with 4′,6-diamidino-2-phenylindole (DAPI) to mark the nuclei (Figs. 4 and S4). All PDOs proliferated in the presence of the dimethyl sulfoxide vehicle control. PDOs #7 and #27 which were isolated from the treatment naïve ER+ papillary carcinoma *in situ* and invasive ductal carcinoma tumors, respectively, showed the least proliferation, and demonstrated decreased proliferation in response to fulvestrant and/or palbociclib. PDO #8, isolated from a metastatic ER+ bone lesion, treated with letrozole and palbociclib, responded to both fulvestrant, palbociclib, and their combination (Figs. 4, *B* and *C* and S4*A*). This was surprising since the patient had relapsed on this combination therapy. Only PDO #10, isolated from ER+ recurrent cancer from ascites fluid, showed increased resistance toward fulvestrant, palbociclib, and their combination (Fig. 4, *B* and *C* and S4*B*). This is not unexpected given that the patient had been heavily treated with multiple therapies at this point (Fig. 4*A*, Table S1). Collectively, these data reveal that PDOs do not always recapitulate the original patient’s drug responses, suggesting that tumor extrinsic factors, such as stromal environment, might contribute to treatment responses, particularly in cases where the PDOs showed sensitivity to ER and CDK4/6 targeting therapies, but the patient relapsed on these therapies.

### CAFs stimulate resistance in ER+ tumor lines and ER+ PDOs

**Figure 5:**
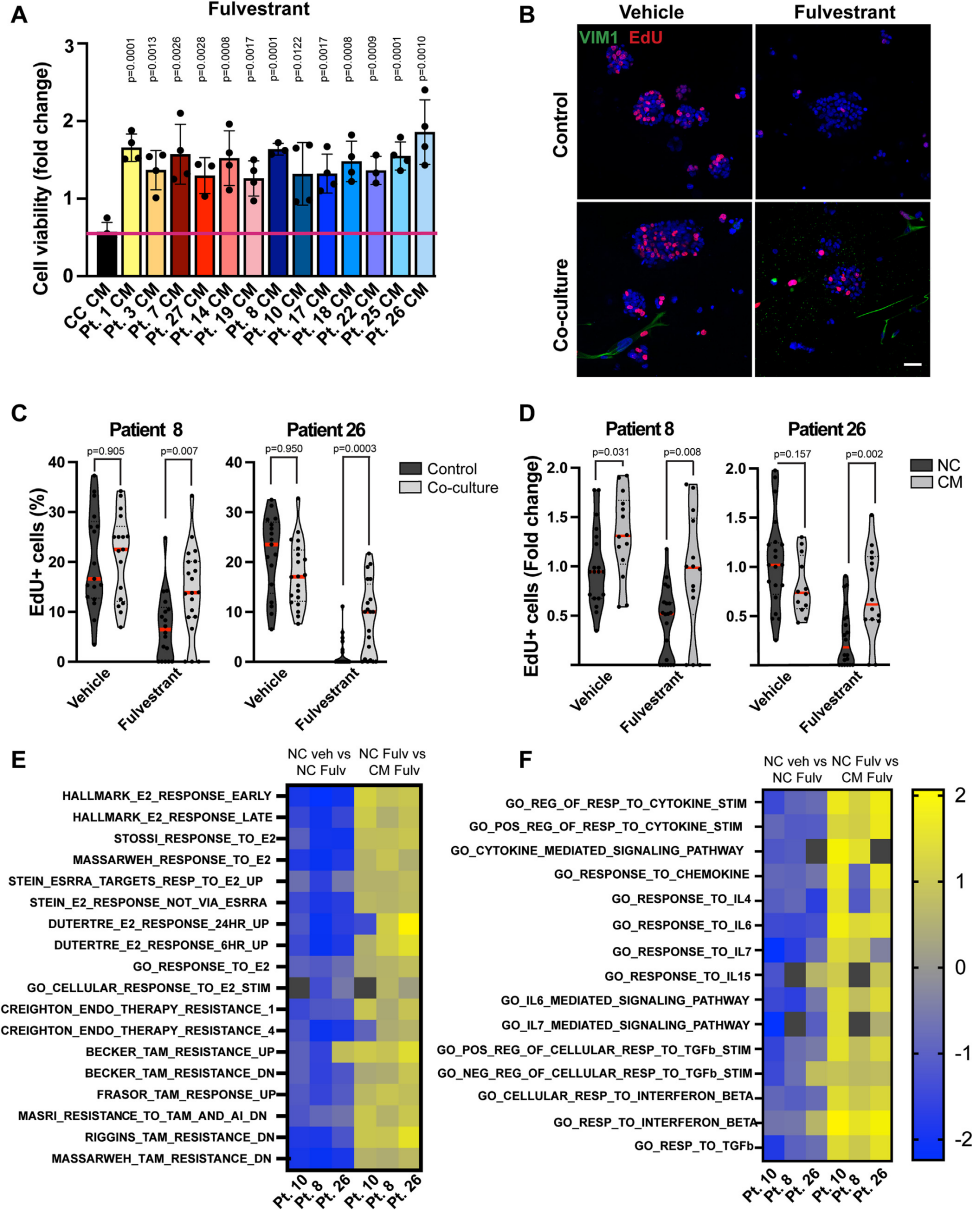
**CAF-conditioned media drives resistance to fulvestrant.***A*, cell viability of MCF7 cells grown in different patient-derived CAF-CM (#1-27) and treated with 500 nM fulvestrant for 72 h. PrestoBlue was used to assess cell viability. Data are presented as mean + SD. Each data point is the mean of one independent experiment. *B*, representative confocal images of control PDOs or PDO-CAF co-cultures stained for VIM1 and EdU. *C* and *D*, quantification of EdU+ (*red*) cells in control PDOs and PDO-CAF co-cultures (*C*) and PDOs grown in nonconditioned or CAF-CM media (*D*), and treated with 500 nM fulvestrant for 96 h. Proliferation was assessed by pulsing PDOs with EdU for 4 h and quantifying the ratio of EdU+ cells per total (DAPI+) number of cells in 20 PDOs. Each data point represents the percentage of EdU+ cells in one PDO, *red line* indicates the mean ratio of EdU+ cells per treatment group. *E* and *F*, heatmap of RNA-seq normalized enrichment scores (NES) for hormone receptor signaling (*E*) and cytokine signaling (*F*) pathways in PDOs grown in nonconditioned media or CAF-CM and treated with 500 nM fulvestrant for 96 h. *Yellow* indicates pathways that were most significantly upregulated and *blue* indicates downregulated pathways (scale: log2). *Black boxes* denote unavailable reads. Student’s *t* test was used to assess significance comparing each CAF-CM to NC sample, and control PDOs to PDO-CAF co-culture. The scale bar represents 40 μm. CAFs, cancer-associated fibroblasts; DAPI, 4′,6-diamidino-2-phenylindole; EdU, 5-ethynyl-2′-deoxyuridine; NC, nonconditioned; PDOs, patient-derived organoids; VIM1, vimentin.

It is known that secreted factors in the TME contribute to treatment responses (23). We therefore speculated that perhaps the patients who had relapsed on multiple therapies, but whose PDOs still responded to all these therapies *in vitro*, might have relapsed due to CAF-secreted factors. We therefore first assessed how conditioned media harvested from our CAF lines isolated from the patient tumors might influence treatment responses to fulvestrant in an established ER+ cell line, MCF7. These data show that CAF-conditioned media from all the CAF lines stimulated resistance toward fulvestrant (Fig. 5*A*). We next assessed how growing PDOs as co-cultures with their matching CAFs or adding CAF-conditioned media would influence the PDOs growth or treatment responses. We treated the PDOs with fulvestrant in the presence and absence of CAFs or CAF-conditioned media harvested from the same tumors as the PDOs. These data revealed that CAFs and media harvested from the matching CAFs significantly stimulated proliferation in the fulvestrant treated PDOs (Figs. 5, *B*–*D* and S5, *A* and *B*). We next investigated whether CAFs ability to induce resistance to fulvestrant is a more general phenomena and tested conditioned media derived from multiple CAF lines in two PDO lines that had not recapitulated the patients’ treatment responses (PDOs #8 and #26 that showed sensitivity *in vitro* although the patient had relapsed on endocrine therapy (Fig. 4). Indeed, these data show that all the tested CAF lines stimulated resistance toward fulvestrant regardless of whether the CAFs were isolated from the same tumors or not (Fig. S5*C*). These data suggest that stromal cells/CAFs can stimulate treatment resistance and might explain cases where the PDOs grown alone do not recapitulate the original patient responses.

To gain insight into the gene expression profile of the PDOs under endocrine therapy, and how the CAFs might influence gene expression and stimulate resistance, we performed RNA sequencing on three of the metastatic PDOs (#8, #10, and #26) treated with control media and CAF-conditioned media, in the presence and absence of fulvestrant (4-days treatment). These data revealed that fulvestrant significantly suppressed the ER gene expression signature in the PDOs, suggesting that the ER-pathway is active in these tumors although the patients had relapsed on ER-targeting agents (tamoxifen and letrozole) (Fig. 5*E*, Tables S2–S4). Furthermore, we observed enrichment of endocrine resistance signatures in PDOs grown in CAF-conditioned media and treated with fulvestrant (Figs. 5*F* and S5*D*, Tables S2–S4). We further discovered that cytokine responsive pathways were highly enriched in fulvestrant-treated PDOs in CAF-conditioned media (Fig. 5*F*, Tables S2–S4), suggesting that CAF-secreted cytokines might contribute to endocrine therapy resistance particularly in the PDOs that had relapsed on endocrine therapy.

### CAF-secreted cytokines stimulate treatment resistance

**Figure 6:**
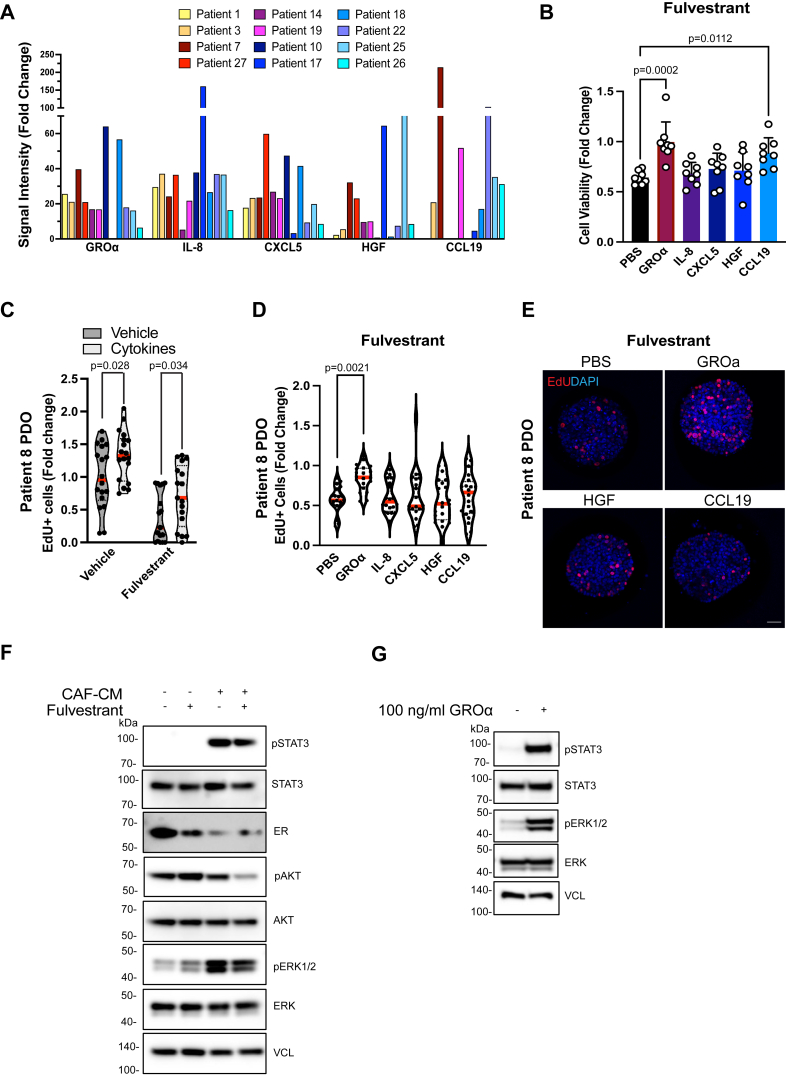
**CAF-secreted cytokines drive resistance to fulvestrant.***A*, quantification of the signal intensity of the five highly secreted cytokines from the cytokine arrays of CAF-CM (patients #1-27) normalized to cancer cell-CM. *B*, cell viability of MCF7 cells treated with recombinant human GROα/CXCL1, IL-8, CXCL5, HGF, CCL19, or PBS (100 ng/ml) and fulvestrant (500 nM). Presto *Blue* was used to assess cell viability after 72 h. Data are normalized to PBS treated groups and is a representative of three independent experiments. Error bars are SEM. *C* and *D*, quantification of EdU+ cells in PDOs treated with a cytokine cocktail and fulvestrant (500 nM) (*C*) or with recombinant human GROα, IL-8, CXCL5, HGF, CCL19, or PBS (100 ng/ml) and fulvestrant (500 nM) from two independent experiments. Proliferation was assessed by pulsing PDOs with EdU for 4 h and quantifying the ratio of EdU+ cells per total (DAPI+) number of cells in 20 PDOs. *Red line* indicates the mean. *E*, representative confocal images of PDOs treated with recombinant human GROα, HGF, CCL19, or PBS (100 ng/ml) and fulvestrant (500 nM). *F*, Western blot analysis of CAF-CM stimulated signaling pathway proteins pSTAT3, STAT3, ER, pAKT, AKT, pERK1/2, and ERK1/2 in PDOs grown in NC or CAF-CM and treated with 500 nM fulvestrant for 96 h. *G*, Western blot analysis of GROα stimulated signaling pathway proteins pSTAT3, STAT3, pERK1/2, and ERK1/2 in MCF7 cells starved overnight and incubated for 30 min with 100 ng/ml GROα. VCL was used as loading control. Student’s *t* test was used to determine significance by comparing each treatment to the PBS control in (*B*) and (*D*). The scale bar represents 40 μm. CAF, cancer-associated fibroblasts; DAPI, 4′,6-diamidino-2-phenylindole; EdU, 5-ethynyl-2′-deoxyuridine; ER, estrogen receptor; GROα, growth-regulated oncogene α; HGF, hepatocyte growth factor; IL, interleukins; PDOs, patient-derived organoids; VCL, vinculin.

Because we observed that CAF-secreted factors drive treatment resistance and that cytokine responsive pathways were upregulated in the CAF-treated PDOs, we next sought to identify some of the cytokines that are highly secreted by our CAF lines that might contribute to the observed fulvestrant resistance. To characterize the cytokine secretion profile of each CAF line, we harvested CAF-conditioned media after 48 h and compared this to media harvested from ER+ tumor cells as a control. We subjected the CAF-CM or cancer-cell conditioned media (CM) to the human XL cytokine array (R&D Systems) that recognizes 105 cytokines. These data revealed multiple CAF-secreted cytokines (Fig. 6*A*) and illustrated significant heterogeneity in the CAFs secretion profiles. For example, some of the cytokines (CCL19 and GROα) were variably expressed between different CAF lines ranging between 15 and 200× higher levels in the CAFs compared to tumor cells. In addition, cytokines that are known to stimulate immunosuppressive environments, such as interleukins (IL)-8, were highly secreted by all the CAF lines (Fig. 6*A*). We next wanted to determine whether any of the highly secreted cytokines might contribute to endocrine treatment resistance. We treated ER+ tumor cells (MCF7) with fulvestrant in the presence or absence of the top five secreted cytokines (GROα/CXCL1, IL-8, CXCL5, hepatocyte growth factor (HGF) and CCL19) (Fig. 6*B*). Although HGF and IL-8 were found to have no effect on resistance against fulvestrant, stimulation with GROα and CCL19 increased tumor cell resistance suggesting that CAF-secreted cytokines can stimulate drug resistance (Fig. 6*B*). Next, we treated PDOs with a cocktail of the top five cytokines. We observed that addition of the five cytokines increase proliferation both in the vehicle-treated conditions as well as in fulvestrant-treated PDOs (Fig. 6*C*). To validate the findings with individual cytokines that we observed with MCF7 cells, we treated the PDOs with fulvestrant in the presence and absence of the highly secreted cytokines and confirmed that GROα significantly induced resistance to fulvestrant. Although CCL19 provided some level of resistance to fulvestrant in the PDOs, there was high variability and thus data did not reach statistical significance (Fig. 6, *D* and *E*). To investigate the downstream signaling pathways the secreted cytokines might activate in the PDOs (40), we grew the PDOs in CAF-CM and treated them with and without fulvestrant. We observed that CAF-CM highly stimulated phosphorylation of STAT3 and ERK1/2 (Fig. 6*F*). Furthermore, we observed reduced expression of ER, suggesting that CAF-CM might break the PDOs reliance of ER signaling (Fig. 6*F*). To validate the signaling changes were due to cytokine signaling, we stimulated tumor cells with GROα cytokine, and again observed increased phosphorylation of STAT3 and ERK1/2 (Fig. 6*G*). Collectively, these data suggest that CAF-secreted cytokines can play a role in treatment resistance and could explain cases where resistance-driving mutations are not found in the relapsed patient tumors.

## Discussion

Our work describes an improved method for the isolation and propagation of ER+ PDOs that has resulted in a higher take-rate of patient matched PDOs while allowing the simultaneous isolation of CAFs from the same biopsies. Historically, HR+ breast cancer models for preclinical testing have been difficult to establish and are limited to a handful of models (41, 42, 43, 44, 45, 46). Furthermore, propagation of PDOs from HR+ metastasis has been difficult, with only 2 to 3 bone metastasis PDxO lines derived to date (18). The method presented here allows more efficient isolation and take-rate particularly for HR+ metastasis, resulting in an overall take-rate of ∼78% for the metastatic samples. Additionally, the majority of HR+ PDOs grown in this study retained their ER expression and ER-responsiveness (assessed by their gene expression and proliferative response to fulvestrant treatment) over prolonged culture periods (>1 year). Our method also provides the advantage of reducing the cost and time required to generate PDOs by circumventing the necessity to passage through a mouse host, which ultimately simplifies the PDO pipeline from the clinic to the laboratory. We speculate that our improved take-rate is partly due to using fresh biopsies, quick sample delivery from the clinic to the processing lab (∼30 min), and sample digestion into smaller fragments instead of single cells. Additionally, we have slightly modified the media formulation originally published by Sachs *et al.* (15), by adding hydrocortisone and 17-beta-estradiol which improved our take-rate particularly from metastases. Specifically, the addition of CXCL12, osteopontin and insulin-like growth factor 1 to the bone metastasis media has improved our take-rate from the bone metastasis samples.

To enhance the probability of the successful propagation of PDOs, most protocols remove the CAFs from tissue samples to prevent them from overtaking the PDO cultures (15, 16, 17, 47). Instead of discarding these CAFs, we have optimized a protocol that acquires monocultures of both, matched patient-derived CAFs and the PDOs. Our protocol has successfully derived CAFs and PDOs by digesting the biopsy fragments of breast tumors and then embedding digested tissue fragments/cell suspension into BME for PDO cultures and plating a small fraction on fibronectin-coated plates for CAF cultures. Several studies have supported the role of CAFs as a critical mediator of disease progression (20, 21, 22, 23, 24); thus, our optimized protocol offers the advantage of enabling the investigation of resistance mechanisms induced by the CAFs. Since CAFs contribute to disease progression and drug resistance, CAFs have been explored as targets for cancer therapy (48). However, targeting CAFs has been challenging due to the heterogeneity of CAF populations. Recently, several subtypes of CAFs were identified in breast cancer (30, 39, 49, 50, 51, 52). While some subtypes promote tumor cell proliferation and invasion, the tumorigenic function of other subtypes is yet to be identified (39). We observed differences in expression levels and combination of fibroblasts markers in our different CAF lines. Our CAF lines are likely to represent a mixture of different subtypes depending on the disease stage and original location of the biopsy. Interestingly, all CAF lines and normal fibroblast were able to stimulate resistance to fulvestrant. It is plausible that the stiff microenvironment of a plastic dish stimulates activation of the normal fibroblasts (53, 54, 55), and therefore, the secretome profile is likely to be slightly different when the cross talk from the 3D microenvironment is lacking. Furthermore, we speculate that our CAF lines consist of heterogenous populations within each line as heterogeneity within CAF lines is expected (30, 39, 49, 50, 51, 52) and encourages additional characterization, such as *via* single cell sequencing approaches. Interestingly recent articles have reported CD146 negative CAFs to downregulate ER expression and stimulate tamoxifen resistance in breast cancer (25, 56). Given that we observed downregulation of estrogen receptor 1 with the addition of CAF-conditioned media, it is possible that these CAFs represent the CD146 negative lineage shown to stimulate more aggressive tumor behavior. Ultimately, the incorporation of CAFs will allow studies that reflect a more accurate representation of the TME and allow studies into how different CAF subpopulations contribute to disease progression.

Numerous studies have highlighted the role of CAFs in advancing tumor progression and treatment resistance (20, 21, 22, 23, 24, 25, 26, 27, 28, 29, 30, 31). This is particularly interesting given our data showing that some of the PDOs isolated from relapsed patients were still sensitive to fulvestrant when cultured alone and only became resistant in the presence of CAF-conditioned media. These data could suggest that tumor extrinsic factors might be responsible for treatment relapse in some of the cases. Therefore, to further investigate the effect of CAFs on treatment resistance, we treated our PDOs with fulvestrant in CAF-CM and performed RNAseq analysis. These data show revealed an upregulation of cytokine responsive pathways in the PDOs cultured in CAF-conditioned media. Since a substantial number of cytokine responsive pathways were upregulated in CAF-CM, we investigated the role of cytokines in treatment resistance further. Our data suggest that multiple cytokines can induce resistance against fulvestrant. Although our studies only investigated the effect of cytokines on the tumor cells and PDOs, it is critical to recognize the role these cytokines may have on regulating tumor immunity. For example, our cytokine array identified IL-8 as the highest-secreted cytokine by our established CAF lines. Many studies have extensively reported the role of IL-8 in contributing to an immunosuppressive environment (57). Therefore, many of the CAF-secreted cytokines likely play a role in generating an immunosuppressive environment that is frequently observed in HR+ breast tumors. Although our protocol cannot assess the secreted cytokines’ effect on immune cells, due to lack of concurrent blood collection, our data do show that the cytokines have tumor specific effects, stimulating proliferation and treatment resistance in the HR+ breast tumors. For example, our study shows GROα stimulating treatment resistance toward endocrine therapy. Interestingly STAT3 and ERK signaling that we observed being stimulated by GROα have been implicated in treatment resistance in HR+ breast cancer (58, 59, 60, 61, 62, 63). In this context, it is intriguing to speculate that these same cytokines might also contribute to the immunosuppressive environment and that targeting these cytokines might have a dual effect on the tumor regression, by targeting tumor cells themselves, as well as enhancing antitumor immunity.

In conclusion, PDOs have tremendous promise, offering a balance of tissue complexity that can mimic native biology while being produced at a relatively lower cost and shorter time frame. The addition of CAFs also adds another layer to tissue complexity by preserving many of the factors present in the TME. This balance of biologic complexity with reduced cost/time for organoid development may also be the key to developing a co-clinical patient-derived model for assessing treatment responses and personalizing clinical care. In an era with increasing drug targets, novel therapeutic options, and rationale for a multitude of combinations, the ability to test multiple approaches in patient-derived models will be instrumental for discovery and translation into clinical testing. This approach would also overcome the difficulties of designing and implementing clinical trials to address patient-specific druggable targets.

## Experimental procedures

### Cell culture of established cell lines

MCF7 cells were a kind gift from Dr Joan Brugge (Harvard Medical School) and authenticated by short tandem repeat analysis. MCF7 cells were tested once a month for mycoplasma using MycoAlert *Mycoplasma* detection kit (Lonza) and grown in Dulbecco's modified Eagle's medium (DMEM) supplemented with 10% fetal bovine serum (FBS) and penicillin/streptomycin at 37 °C with 5% CO2.

### Drug testing and cell viability assays

To make conditioned media, MCF7 cells or CAFs were grown in DMEM supplemented with 1% FBS, and conditioned media was harvested and filtered 48 h later. MCF7 cells were seeded at 12,000 cells/well on a 96-well plate. The following day media was replaced with cancer cell–conditioned media or CAF-conditioned media and 500 nM fulvestrant (Selleckchem #S1191) was added. For cytokine treatment, cells were stimulated with 100 ng/ml of recombinant human GROα, IL-8, CXCL5, HGF, CCL19, or PBS and cytokines were readded after 48 h (PeproTech). Cell viability was assessed after 72 h using PrestoBlue HS cell viability reagent (Invitrogen).

### Patient screening and data collection

The institutional review board (IRB) protocol was reviewed by the Dana-Farber/Harvard Cancer Center Scientific Review Committee and IRBand approved in 2017 (#17-627). The tissue collection study was set up for patients evaluated for disorders affecting the breast. Only patients with a breast lesion with high suspicion of breast origin requiring a diagnostic/therapeutic procedure as part of standard of care were enrolled. In addition, patients with active breast cancer were also invited to participate. Informed consent was obtained from all participants as per Federal Regulations (45 CFR 46), BIDMC IRB Guidelines and requirements of Health Insurance Portability and Accountability Act, and the studies abide by the Declaration of Helsinki principles.

Screening for breast masses occurred through standard diagnostic protocol of mammography, ultrasound and/or MRI of breasts. Screening of metastasis occurred through CT imaging with contrast, [^18^F] Fluorodeoxyglucose PET/CT or MRI. The criteria to enroll were (1) at least 18 years of age, (2) target tissue either located in the breast or of high suspicion for breast origin (*i.e.* metastatic site or pleural fluid in patient with history of breast cancer), (3) target lesion must have at least one measured axis be greater than or equal to 1.75 cm, (4) each of the other two axis must be greater than 0.5 cm, (5) ability to understand and the willingness to sign a written informed consent document, and (6) Planned biopsy as part of standard of care. We also collected data regarding patient demographics, pathology results for diagnostic biopsy and/or surgical resection, molecular and genomic findings, clinical stage, treatment plan, and response/recurrence. All personal identifiers were removed before the biopsies were transferred to the research laboratory, and the samples were given unique identifier codes.

### Propagation of PDOs

Breast tissue was collected through either a CNB or FNA biopsy, and at least 2 to 4 CNB or FNAs were collected. CNB or FNA were placed in cold PBS containing penicillin–streptomycin and 25 mM glucose and transported to the research laboratory within 15 to 30 min. CNB or FNA were cut into small fragments and digested in 1x Dispase-II solution (#Sigma-Aldrich, SCM133) supplemented with 2 mg/ml collagenase I and 5 μM of Y-27632 in an orbital shaker for 45 to 60 min at 37 °C. The digested tissue was sequentially sheared by vigorously pipetting up and down 10 to 20 times using 10 ml and 5 ml plastic pipettes, and the collected fractions were strained over a 100 μm filter. Cell cluster fractions were collected, 5% FBS was added to the suspension, and the fractions were pelleted by centrifugation at 400*g* for 5 min. The cell fractions were embedded into Cultrex growth factor-reduced BME type II (Trevigen 3533-001-02), 50 μl drops were plated into a 24-well plate, and 500ul PDO media was added 30 min later. Any pleural fluid samples were washed and embedded into BME.

### Media optimization and maintaining PDOs

To optimize the media for support of HR+ PDO growth, we tested two modified media. Modified media #1 contained Advanced DMEM/F12 (Gibco) supplemented with 5 ng/ml FGF7, 20 ng/ml FGF10, 5 ng/ml EGF (all from PeproTech), 500 nM TGFBRII inhibitor A82-01 (Tocris #2939), 500 nM p38 MAPK-inhibitor SB202190 (Selleckchem, #S1077), 500 uM N-acetylcysteine, 1 mM nicotinamide, 0.5 ng/ml 17β-estradiol and 50 μg/ml Primocin. Modified media #2 contained Advanced DMEM/F-12 supplemented with 200 ng/ml R-spondin 3, 5 nM neuregulin, 80 ng/ml noggin, 5 ng/ml FGF7, 20 ng/ml FGF10, 5 ng/ml EGF (all from PeproTech), 500 nM TGFBRII inhibitor A82-01 (Tocris #2939), 500 nM p38 MAPK-inhibitor SB202190 (Selleckchem, #S1077), 1X B27 supplement, 500 uM N-acetylcysteine, 1 mM nicotinamide, 50 ng/ml hydrocortisone, 0.5 ng/ml 17β-estradiol, 50 μg/ml primocin. To support growth of bone metastasis samples, the modified media #2 was supplemented with 10 ng/ml CXCL12, 20 ng/ml IGF-1, and 10 ng/ml Osteopontin (all from Peprotech). PDO growth was assessed before passaging and after passages one and two by measuring the diameter using CellProfiler software (https://cellprofiler.org) (64) or by analyzing cell proliferation incubating PDOs with 10 μM EdU for 4 h. For the first 5 days, all media were supplemented with 5 μM of Y-27632. Media were replaced every 4 days, and PDOs passaged every 1 to 3 weeks at 1:2 to 1:4 ratio. For passaging, PDOs were incubated in 1× Dispase-II solution with 2 mg/ml collagenase for 30 to 45 min at 37 °C, and mechanically disrupted by passing through a 26G needle. PDO cultures were confirmed to be negative for CAFs by vimentin staining.

### Propagation of and culturing CAFs

CAFs were propagated from same CNB or FNA as the PDOs. Following tissue digestion, a small fraction of the fragments was plated onto fibronectin coated plates. CAFs were grown in human fibroblast expansion media (Gibco M106500) supplemented with low serum growth factor kit (Gibco S003K) and 4% FBS. CAFs were passaged every 4 to 6 days at 1:2 to 1:4 ratio. For immunofluorescence staining, CAFs were plated onto poly-L-lysine coated coverslips.

### Assessing drug sensitivity in PDOs

To analyze drug sensitivity, PDO fragments were plated into 4-well or 8-well chamber slides at 200 to 600 fragments/well and 500 nM fulvestrant and/or 1uM palbociclib was added the following day. PDOs of early passages (passage 5–8) were used to characterize drug sensitivity. To make conditioned media, CAFs were grown in empty advanced DMEM/F12 media, and conditioned media were harvested 48 h later and mixed 1:1 with completed PDO media. To assess the effect of CAFs on drug resistance, 2000 to 6000 CAFs were plated around a dome containing PDOs in BME, or PDOs were grown in nonconditioned and CAF-CM. For cytokine treatment, PDOs were stimulated with 100 ng/ml of recombinant human GROα, IL-8, CXCL5, HGF, CCL19 (all from PeproTech), or PBS and spiked at 48 h. Cell proliferation was assessed after 96 h of treatment by incubating PDOs with 10 μM -EdU for 4 h.

### Whole-mount staining

PDOs were grown in 4-well chamber slides (Falcon), fixed with 4% paraformaldehyde (PFA) for 30 min, washed with PBS, and permeabilized with wash buffer (0.3% Triton X-100 in PBS) for 20 min. EdU labeling was performed for 40 min using the EdU Click-IT imaging kit (Invitrogen) according to the manufacturer’s description. PDOs were washed three times with wash buffer, blocked for 1 h with blocking buffer (5% goat serum, 0.2% *bovine serum albumin*, 0.3% Triton X-100 in PBS), and incubated with anti-ER antibody (Abcam #ab16666), anti p21 antibody (CST #2947), or anti-Ki67 (Agilent #M7240) overnight at 4 °C in blocking buffer. The following day the PDOs were washed ten times with wash buffer, incubated with secondary antibody (Alexa Fluor 488 or Alexa Fluor 594) for 2 h at room temperature (RT), washed extensively with wash buffer and mounted using Vectashield mounting media containing DAPI (Vector Laboratories). PDOs were imaged with Zeiss LSM 880 confocal microscope. To assess proliferation, 20 PDOs were imaged, and the ratio of EdU positive cells per total number of cells was quantified. Statistical analysis was performed with Prism GraphPad (https://www.graphpad.com/features). Student’s *t* test was used to calculate *p*-values.

### PDO embedding and immunohistochemistry

PDOs were pooled from 3 to 5 wells and incubated for 1.5 h on ice in cell recovery buffer. PDOs were washed with PBS, spun down at 500 rpm for 3 min, and then fixed in 4% PFA for 30 min. Fixed PDOs were stained for 5 min with hematoxylin, washed with water and resuspended in warm Histogel (Epredia HG400001). The histogel and PDO mix was moved to an insert with 200 μl cooled histogel and placed on ice for 15 min. Solidified histogel containing the PDOs was placed in a cassette, fixed overnight in 10% formalin and embedded into paraffin. After deparaffinization, the sections were treated with low pH citrate buffer in a microwave at full power for 2 min and then 10 min at low power. Immunohistochemistry for estrogen receptor (Abcam #ab16666) was performed using the ImmPRESS Excel Amplified Polymer Staining kit (Vector laboratories #MP-7601) according to the manufacturer’s description. The sections were imaged using an Olympus brightfield microscope and scored by a trained pathologist for nuclear features and ER staining.

### Immunofluorescence staining

CAFs were fixed with 4% PFA for 10 min, washed with PBS, and blocked and permeabilized with blocking buffer (1% *bovine serum albumin*, 0.1% Triton X-100 in FBS) for 15 min. CAFs were incubated with anti-alphaSMA (Abcam #5694) diluted in blocking buffer overnight at 4 °C, washed with PBS, incubated with secondary antibodies (Alexa 488) for 1 h at RT and mounted using VectaShield mounting media containing DAPI. CAFs were imaged with Zeiss LSM 880 confocal microscope.

### Western blot

PDOs were pooled from six wells and incubated for 1.5 h on ice in Cell Recovery Buffer supplemented with phosphatase inhibitors (Roche #4906845001). PDOs and CAFs were lysed using RIPA buffer (Boston BioProducts #BP-115) containing protease and phosphatase inhibitors (Thermo Fisher Scientific #A32959) and then centrifuged at 13,000 rpm for 10 min at 4 °C. Protein concentrations were measured using Pierce bicinchoninic acid protein assay kit and then boiled in 4x lithium dodecyl sulfate sample buffer (Invitrogen NP0007). Proteins (20 μg per sample) were separated by electrophoresis on SDS-polyacrylamide gels (Invitrogen # NP0321BOX or XP04200BOX) and transferred to polyvinylidene difluoride membranes at 25 V overnight at 4 °C. The membranes were blocked with 5% nonfat milk in 0.1% Tween 20 in Tris-buffered saline for 1 h and incubated with primary antibodies for 1 h in RT or overnight at 4 °C using the following antibodies: FN1 (Thermo Fisher Scientific #MA5-11981), PDGFRα (CST #3174), VIM (CST #5741), αSMA (Abcam #5694), CAV1 (CST #3267), FAP (CST #66562), PDPN (CST #9047), THY1 (CST #13801), pSTAT3^Tyr705^ (CST#9145), STAT3 (CST#9139), ER (CST #13258), pAKT^Ser473^ (CST#4060), AKT (CST #9272), pERK1/2^Thr202/Tyr204^ (CST #4370), ERK1/2 (CST #4695) and VCL (CST #13901). The membranes were then incubated with appropriate *horseradish peroxidase*-conjugated secondary antibodies (CST #7074 or Invitrogen #32430) for 1 h. Enhanced chemiluminescence was performed using Pierce SuperSignal West Femto Maximum Sensitivity Substrate (Thermo Fisher Scientific #PI34096) and visualized using Amersham Imager 600. Membranes were stripped for 10 min using 20 mM NaOH and reblotted one time.

### Cytokine array

CAFs and MCF7 cells (as a control) were plated on 10 cm dishes in 10% media. When the cells had reached 80% confluency, media was changed to 1% serum and harvested 48 h later. CAF-CM and MCF7-CM were sterile filtered through 0.22 μm filter and snap frozen in liquid nitrogen or used as fresh. The frozen or fresh CAF-CM and MCF7-CM were then subjected to the Human XL Cytokine Array Kit (R&D ARY022B) according to the manufacturer’s protocol. Quantification of the signal produced by the amount of analyte bound was conducted using Fiji and the data were normalized to MCF7-CM.

### Exome sequencing

Patients #8, #10, and #26 PDOs were lysed, and DNA was isolated using DNeasy Blood & Tissue kit according to the manufacturer’s instructions. Whole exome sequencing was performed using DNBSEQ at BGI Genomics (https://www.bgi.com/global/science-detail/whole-exome-sequencing). Libraries were prepared using the Agilent V_6 exome kit and subjected to paired-end 150 bp sequencing. After quality control filtering was performed with SOAPnuke, v2.1.0 (65), reads were aligned to the hg38 human genome using BWA, V0.7.17 (https://github.com/lh3/bwa) (66). Variant calling was performed using GATK HaplotypeCaller in GVCF mode (67).

### RNA sequencing

Four replicates of patient #8 and #10 PDOs and two replicates of patient #26 PDOs grown in breast cancer organoid media or CAF-CM spiked with 500 nM fulvestrant were lysed in guanidine isothiocyanate buffer, and RNA was isolated using the RNeasy Fibrous Tissue Mini Kit according to the manufacturer’s instructions. Prior to library preparation, the quality of RNA was assessed with Bioanalyzer (Agilent Technologies). The mRNA libraries were generated by BGI and bulk RNA sequencing was performed using BGISEQ. Paired-end 100 bp reads were aligned to the GRCh38 human genome using Star 2.7.0f (68). Differential expression was assessed using edgeR, v3.30.3 (69). Differential expression results were ranked by log10(pvalue)∗ sign of logFC and subjected to Gene Set Enrichment Analysis using the fgseaMultilevel function of the fgsea package (v 1.21.2). Analysis was performed in R v4.0.1. RNA sequencing data has been deposited to GEO with access number GSE216540.

### Statistical analysis

Statistical analysis was performed using Prism GraphPad software. All experiments with MCF7 cells were repeated at least twice with 6 to 8 technical replicates. Experiments with PDOs were performed once unless stated otherwise in the figure legend and 15 to 20 PDOs were quantified for each experiment. Data are presented as mean ± SD. Student’s *t* test was used to assess significance.

## Data availability

Data included in this manuscript are included in the manuscript, figures, and supplemental files (figures and tables). For the RNAseq the data are deposited to GEO with access number GSE216540. The exome-sequencing raw data are not shared due to patient confidentiality and per our IRB agreement.

## Supporting information

This article contains supporting information.

## Conflict of interest

The authors declare that they have no conflicts of interest with the contents of this article.
