# Supplementary material for: Simultaneous isolation of hormone receptor–positive breast cancer organoids and fibroblasts reveals stroma-mediated resistance mechanisms
Source: J Biol Chem. 2023 Jul 7;299(8):105021. doi: 10.1016/j.jbc.2023.105021 (PMC10415704; doi:10.1016/j.jbc.2023.105021)
Supplement: Supporting Figure S1 [file mmc5.pdf]

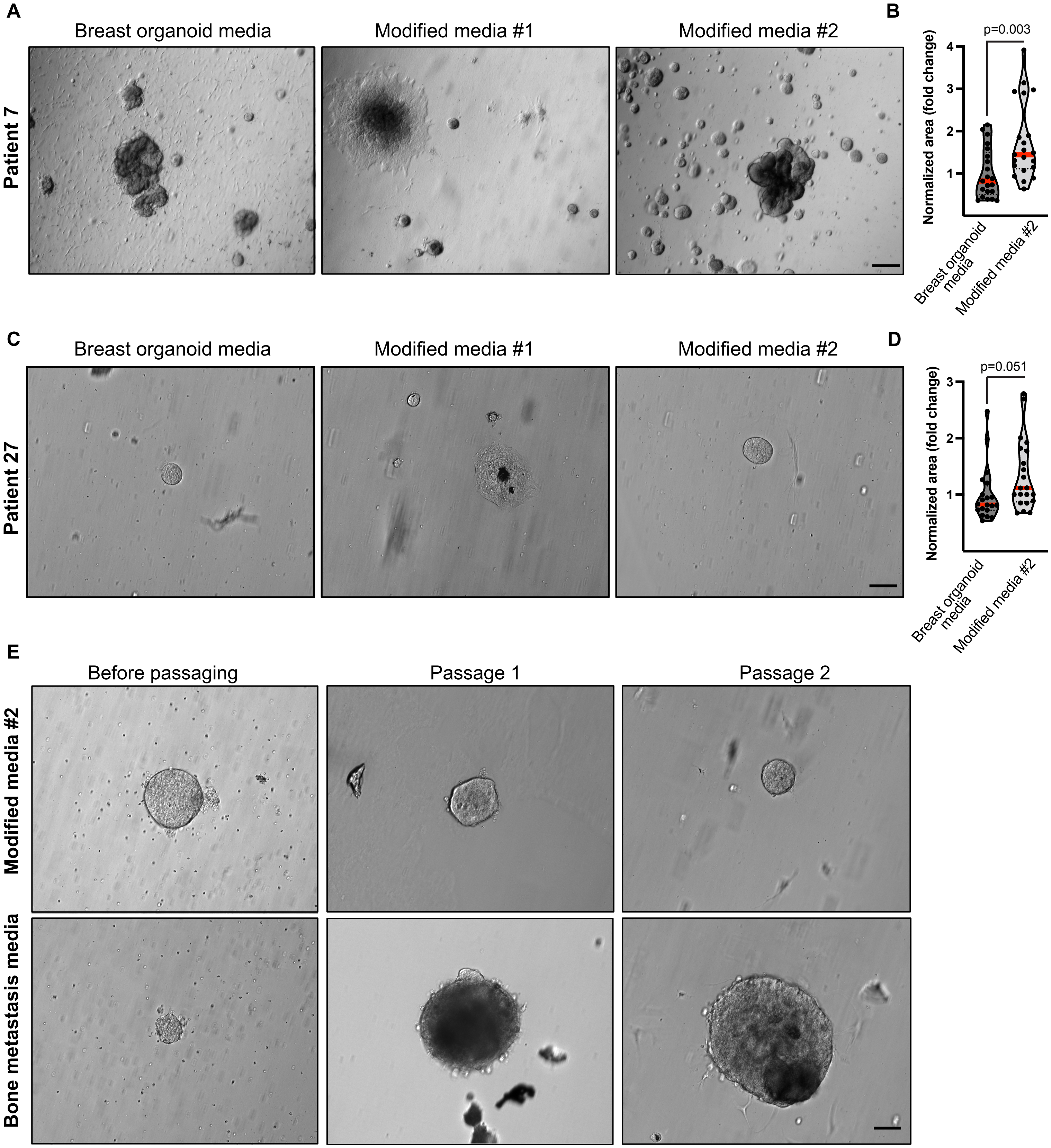

Figure S1. Optimized media supports growth of HR+ patient-derived organoid cultures. A) Brigt-field images of Patient #7 PDOs (papillary carcinoma in situ) grown in published breast organoid media, modified media #1 and modified #2. B) Quantification of diameter in PDOs grown in organoid media and modified media #2 at passage 2. C) Brigtfield images of Patient #27 PDOs (invasive ductal carcinoma) grown in published breast organoid media, modified media #1 and modified #2. D) Quantification of diameter in PDOs grown in published breast organoid media and modified media #2 at passage 2. Red line indicates the mean diameter of each treatment group E) Brightfield images of Patient #8 PDOs (bone metastasis) before passaging, at passage 1 and passage 2 in modified #2 or bone metastasis media. Student's T-test was used to assess significance. Scalebars: 500 um (A), 200 um (C, E)
