# Supplementary material for: Simultaneous isolation of hormone receptor–positive breast cancer organoids and fibroblasts reveals stroma-mediated resistance mechanisms
Source: J Biol Chem. 2023 Jul 7;299(8):105021. doi: 10.1016/j.jbc.2023.105021 (PMC10415704; doi:10.1016/j.jbc.2023.105021)
Supplement: Supporting Figure S2 [file mmc6.pdf]

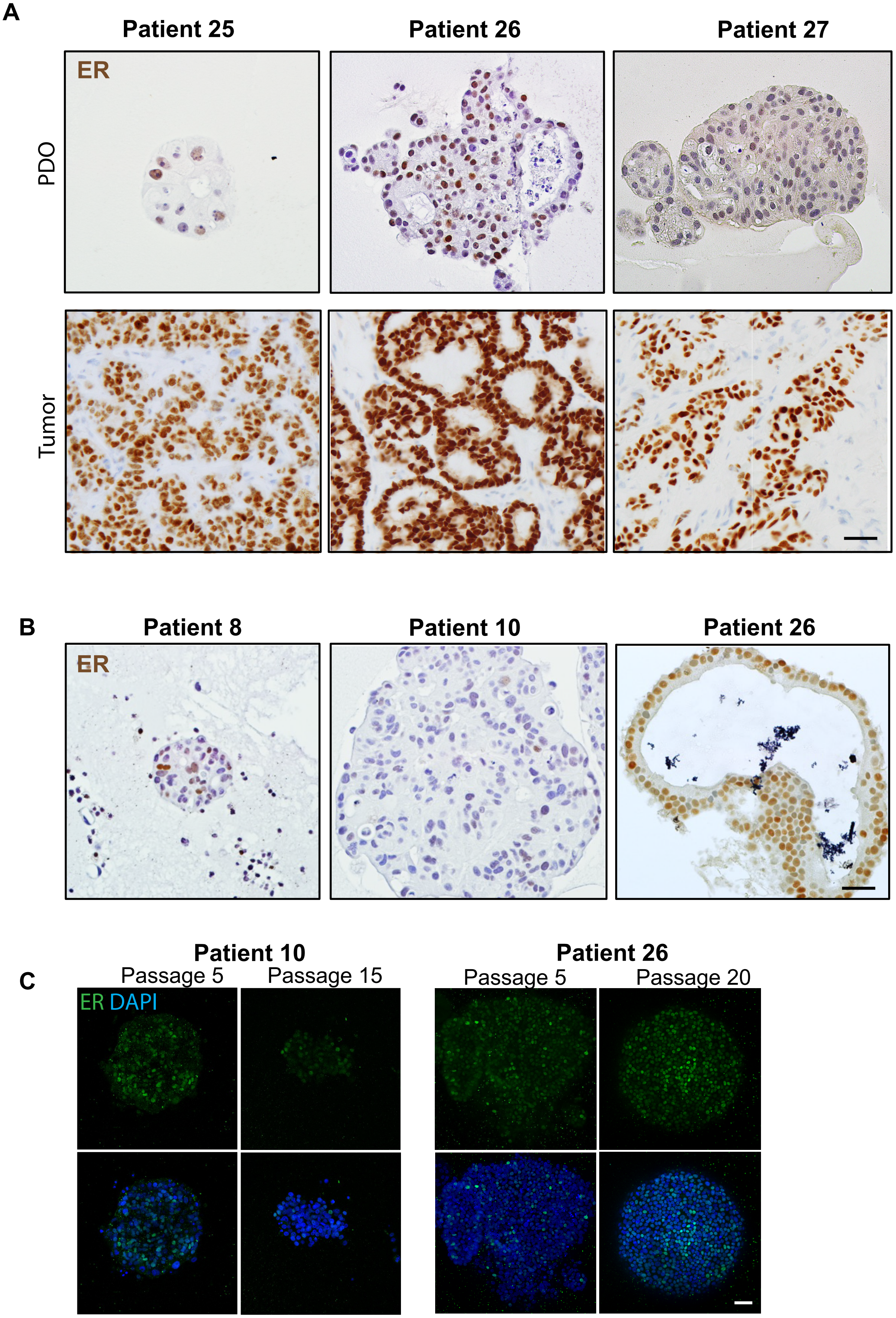

Figure S2. Patient-derived organoid cultures retain Estrogen Receptor (ER) expression during long-term culture. A) ER immunohistochemistry of matching PDOs and patient tumors. B) ER immunohistochemistry of patient #8, #10 and #26 PDOs at passage 20 C)Representative confocal images of patient #10 and #26 PDOs stained for ER (green) and nuclei (DAPI: blue) at passage 5 and passage 15 or 20. Scale bars: 50 um (A, B), 40 um (C).
