# Supplementary material for: Simultaneous isolation of hormone receptor–positive breast cancer organoids and fibroblasts reveals stroma-mediated resistance mechanisms
Source: J Biol Chem. 2023 Jul 7;299(8):105021. doi: 10.1016/j.jbc.2023.105021 (PMC10415704; doi:10.1016/j.jbc.2023.105021)
Supplement: Supporting Figure S3 [file mmc7.pdf]

Figure S3.

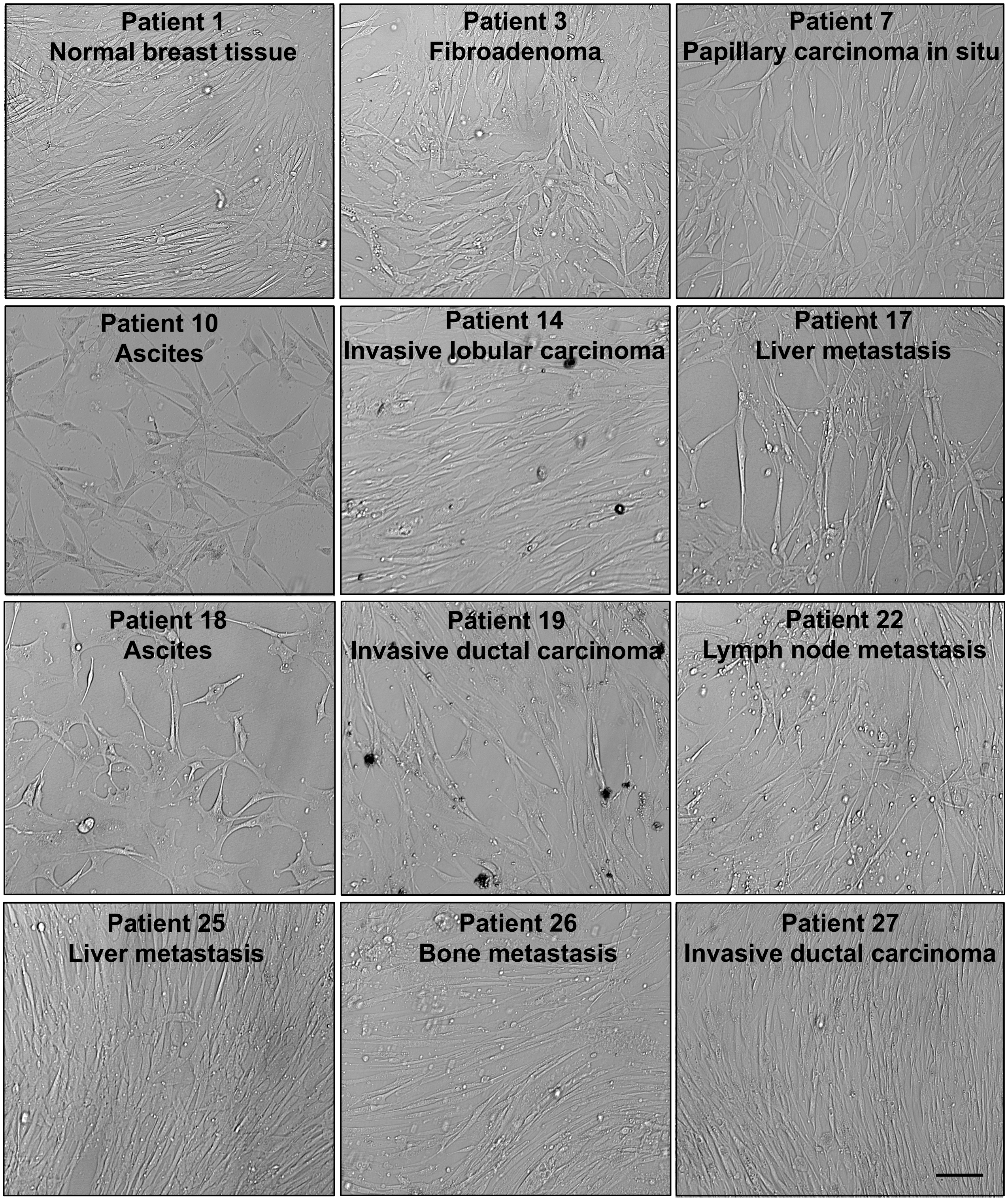

Figure S3. Propagation of patient-derived cancer-associated fibroblasts from primary and metastatic ER+ breast cancer. Brightfield microscopy images of the CAF lines isolated from different patient biopsies. Scale bars: 200 um
