# Supplementary material for: Simultaneous isolation of hormone receptor–positive breast cancer organoids and fibroblasts reveals stroma-mediated resistance mechanisms
Source: J Biol Chem. 2023 Jul 7;299(8):105021. doi: 10.1016/j.jbc.2023.105021 (PMC10415704; doi:10.1016/j.jbc.2023.105021)
Supplement: Supporting Figure S4 [file mmc8.pdf]

Figure S4.

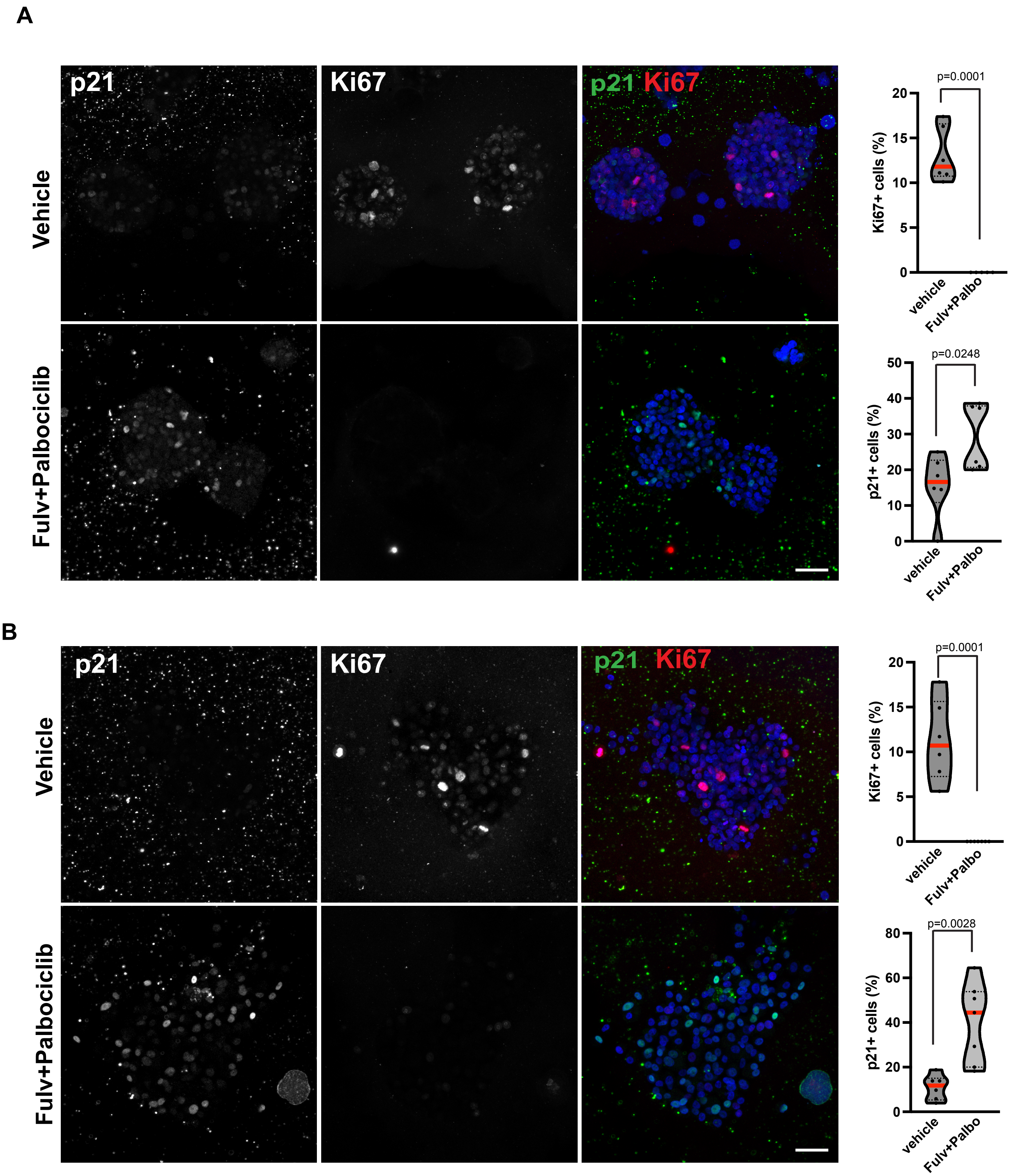

Figure S4. Targeted therapies decrease proliferation of metastatic patient-derived organoid cultures. A) Representative confocal images of p21, Ki67 and DAPI staining and quantification of p21+ and Ki67+ cells in patient #8 PDOs (A) and patient #10 PDOs (B) treated with 500 nM Fulvestrant and 1 uM Palbociclib for 96h. Student's t-test was used to assess significance. Scale bar 40 um.
