# Supplementary material for: Simultaneous isolation of hormone receptor–positive breast cancer organoids and fibroblasts reveals stroma-mediated resistance mechanisms
Source: J Biol Chem. 2023 Jul 7;299(8):105021. doi: 10.1016/j.jbc.2023.105021 (PMC10415704; doi:10.1016/j.jbc.2023.105021)
Supplement: Supporting Figure S5 [file mmc9.pdf]

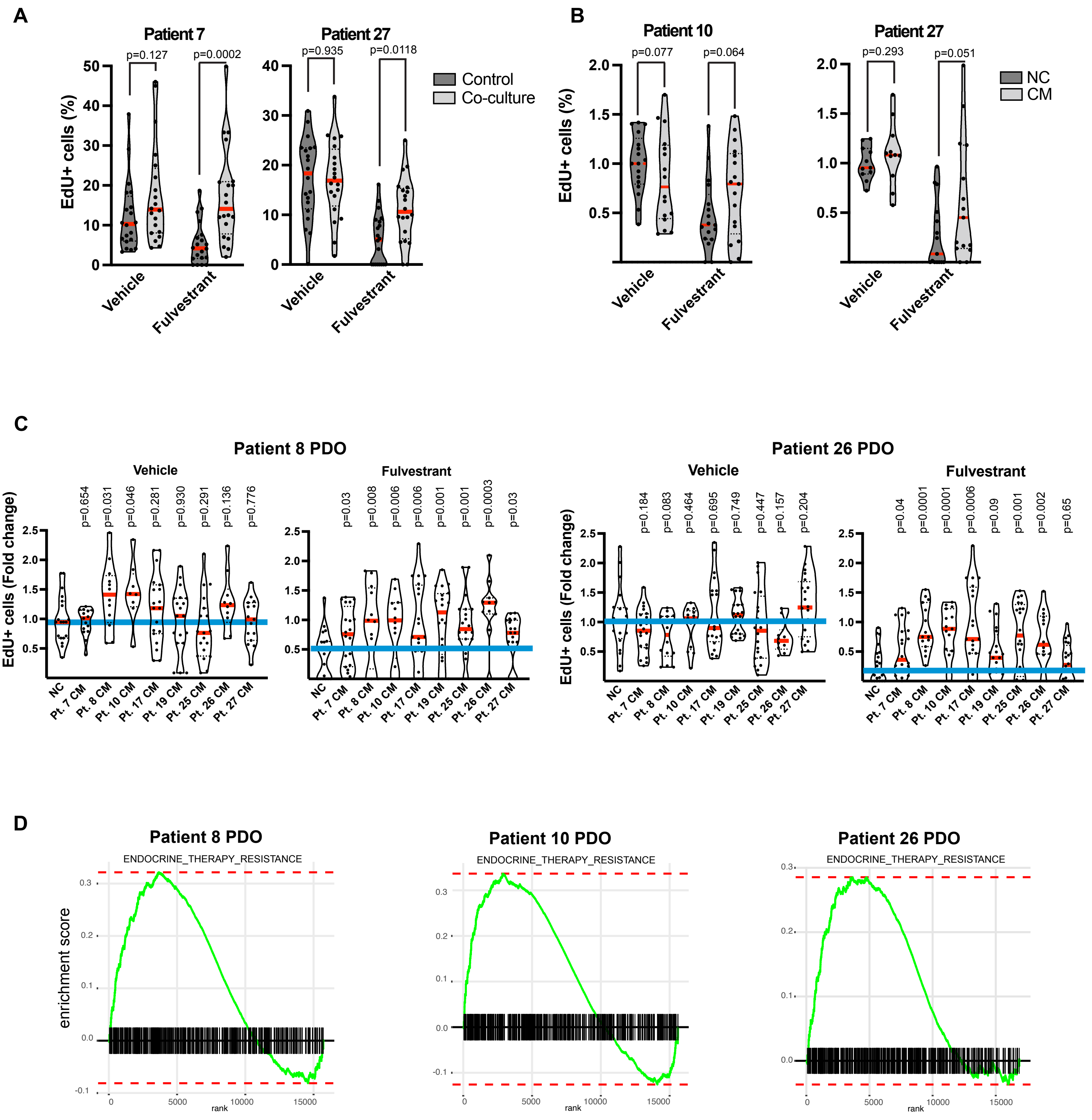

Figure S5. CAF conditioned media drives resistance to Fulvestrant. A-C) Quantification of EdU+ (red) cells in control PDOs or PDO-CAF co-culture (A), and PDOs grown in non-conditioned or CAF-CM media (B-C) and treated with 500 nM Fulvestrant for 96h. Proliferation was assessed by pulsing PDOs with EdU for 4h and quantifying the ratio of EdU+ cells per total (DAPI+) number of cells in 20 PDOs. Each data point represents the percentage of EdU+ cells in one PDO, red line indicates the mean ratio of EdU+ cells per treatment group. Each CM group was normalized to NC control. Student's T-test was used to assess significance comparing each CM to NC control. D) Endocrine therapy resistance gene-set enrichment analysis comparing PDOs grown in CAF CM to PDOs grown in NC, and treated with Fulves-trant.
